# Articular Joint Lubricants during Osteoarthritis and Rheumatoid Arthritis Display Altered Levels and Molecular Species

**DOI:** 10.1371/journal.pone.0125192

**Published:** 2015-05-01

**Authors:** Marta Krystyna Kosinska, Taryn E. Ludwig, Gerhard Liebisch, Ruiyan Zhang, Hans-Christian Siebert, Jochen Wilhelm, Ulrich Kaesser, Reinhard B. Dettmeyer, Heiko Klein, Bernd Ishaque, Markus Rickert, Gerd Schmitz, Tannin A. Schmidt, Juergen Steinmeyer

**Affiliations:** 1 Orthopedic Research Laboratories, Department of Orthopedics, Justus-Liebig-University, Giessen, Germany; 2 Faculty of Kinesiology & Biomedical Engineering Graduate Program, University of Calgary, Alberta, Canada; 3 Department of Clinical Chemistry and Laboratory Medicine, University Hospital, Regensburg, Germany; 4 RI-B-NT Research Institute of Bioinformatics and Nanotechnology, Kiel, Germany; 5 Zoological Institute, Structural Biology, Center for Biochemistry and Molecular Biology, Christian-Albrechts-University, Kiel, Germany; 6 Medical Clinic II/IV, Justus-Liebig-University, Giessen, Germany; 7 Internistisches Praxiszentrum am Krankenhaus Balserische Stiftung, Giessen, Germany; 8 Institute of Forensic Medicine, Justus-Liebig-University, Giessen, Germany; SERGAS (Servizo Galego de Saude) and IDIS (Instituto de Investigación Sanitaria de Santiago), the NEIRID Lab, Research Laboratory 9, Santiago University Clinical Hospital. Santiago de Compostela, SPAIN

## Abstract

**Background:**

Hyaluronic acid (HA), lubricin, and phospholipid species (PLs) contribute independently or together to the boundary lubrication of articular joints that is provided by synovial fluid (SF). Our study is the first reporting quantitative data about the molecular weight (MW) forms of HA, lubricin, and PLs in SF from cohorts of healthy donors, patients with early (eOA)- or late (lOA)-stage osteoarthritis (OA), and patients with active rheumatoid arthritis (RA).

**Methods:**

We used human SF from unaffected controls, eOA, lOA, and RA. HA and lubricin levels were measured by enzyme-linked immunosorbent assay. PLs was quantified by electrospray ionization tandem mass spectrometry. Fatty acids (FAs) were analyzed by gas chromatography, coupled with mass spectrometry. The MW distribution of HA was determined by agarose gel electrophoresis.

**Results:**

Compared with control SF, the concentrations of HA and lubricin were lower in OA and RA SF, whereas those of PLs were higher in OA and RA SF. Moreover, the MW distribution of HA shifted toward the lower ranges in OA and RA SF. We noted distinct alterations between cohorts in the relative distribution of PLs and the degree of FA saturation and chain lengths of FAs.

**Conclusions:**

The levels, composition, and MW distribution of all currently known lubricants in SF—HA, lubricin, PLs—vary with joint disease and stage of OA. Our study is the first delivering a comprehensive view about all joint lubricants during health and widespread joint diseases. Thus, we provide the framework to develop new optimal compounded lubricants to reduce joint destruction.

## Introduction

Lubrication of cartilage within synovial joints entails a complex interaction of several mechanical and molecular factors, resulting in decreased friction between opposing surfaces of articular cartilage. In healthy weight-bearing joints, a layer of lubricating molecules covers the surfaces of articular cartilage and acts as a boundary lubricant, effecting nearly frictionless motion of joints [1, 2]. The lubricant components of synovial fluid (SF), such as hyaluronic acid or hyaluronan (HA) [1], lubricin [3], and surface-active phospholipids [4], interact with and adsorb to the surface of articular cartilage and have been suggested, independently or in combination, to promote boundary lubrication [2]. Alterations in the composition and concentration of these molecules leads to insufficient boundary lubrication and thus might be associated with degenerative joint diseases, such as osteoarthritis (OA) [5–8].

HA is an extracellular matrix component in SF, cartilage, eye fluid, vitreous humor, and lung, kidney, brain, and muscle tissues [9–11]. This glycosaminoglycan has a high molecular weight (MW) distribution in human SF, ranging from 27 kDa to 10 MDa [12–14]. HA forms long nonsulfated chains of repeating disaccharides, comprising D-glucuronic acid and N-acetyl D-glucosamine, and provides SF with its high viscosity [9–12, 15, 16].

Several functions have been attributed to HA such as tissue hydration, lubrication, and interactions with proteins and proteoglycans of the extracellular matrix were found. Also, HA binds to the cell surface receptors CD44 and RHAMM, which mediate signaling pathways in inflammation, cell and tissue functions, and expression of catabolic enzymes, such as aggrecanases [11, 13, 16–18]. HA in the MW range of 0.5–1.0 x 10(6) Da partially restores SF rheological properties and fibroblast-like synoviocytes (FLSs) metabolism in animal models [13]. High-MW HA inhibits phospholipase A_2_ activity and thus protects phospholipid integrity [19].

Notably, HA in SF from 24 human knee joints with advanced OA was found to be shifted toward the low-MW forms, due to enzymatic cleavage of HA chains [6, 20]. In contrast, the MW distribution of HA was unaltered in SF from 5 patients with advanced OA versus 5 patients who were undergoing meniscectomy or ligament reconstruction without any evident OA [12]. The concentration of HA in human OA SF was reported to be normal [12, 20] but declines in RA [6]. Although the specific contribution of HA to overall cartilage boundary lubrication remains a topic of debate, viscosupplementation with intra-articular HA is still used often to treat OA [21, 22].

Lubricin is a large mucin-like glycoprotein that comprises 3 domains: a cysteine-rich, somatomedin B-like N-terminal domain; a mucin-like *O*-linked oligosaccharide-rich domain; and a C-terminal domain [23]. Homologs of lubricin—superficial zone protein, proteoglycan 4 (PRG4), megakaryocyte-stimulating factor precursor, and arthritis-like camptodactyly-arthropathy-coxa vara-pericarditis syndrome protein—are all encoded by *PRG4* [24].

Lubricin is synthesized and secreted by chondrocytes from the superficial zone of articular cartilage, FLSs and cells in the meniscus [24, 25] and is present in SF, where it acts as a cartilage boundary lubricant, alone or synergistically with HA [1, 26]. Lubricin is expressed in human SF as various isoforms [27], the form that provides better boundary lubrication is unknown. SF from human knee joints with advanced OA was reported to have markedly elevated lubricin concentrations compared with normal human SF [8, 20] and a similar friction-lowering lubricating function [8, 20]. However, compared to normal the levels of lubricin can also be low in some OA SF concomitant with a diminished cartilage boundary lubricating function [7]. Lubricin has been proposed to be chondroprotective, potentially protecting articular chondrocytes against apoptosis [28].

The surface of articular cartilage was described to be hydrophobic, most likely due to the hydrophobic hydrocarbon layer that is generated by surface-active phospholipids that have attached [29, 30]. Most phospholipid species possess a zwitterionic head group, harboring a negative charge on the phosphate group and a positive charge on the amine group. Water-insoluble phospholipids was reported to contribute to a small fraction (~11%) of lubricin in SF [4]. FLSs are believed to be a biosynthetic source of phospholipids [31]; other phospholipids can diffuse from the plasma to SF or be released during cell necrosis. Our recently published lipidomic study demonstrated significantly elevated concentrations of most phospholipid species in OA and RA SF [5]. Changes in the relative distribution and levels of certain phospholipids might alter the lubricating ability of SF and modulate synovial joint inflammation [5]. However, the paucity of detailed data on the biological functions of lipid species in RA and OA underscore the necessity for further studies.

HA, phospholipids, and lubricin have been examined *in vitro* individually and in combination, and all of them mediate boundary lubrication in SF [1, 2]. Nevertheless, the SF lubricant that contributes most to boundary lubrication in healthy, OA, and RA joints remains debated [32–35]. Individual differences in the level and composition of lubricants might cause patient-specific variability in joint lubrication and thus the speed of joint deterioration.

No data exist on the collective levels of HA, lubricin, and phospholipids in SF in patients with common joint diseases, such as OA and RA. Thus, the aim of our study was to quantify lubricin, the various molecular weight forms of HA, and all phospholipid species in parallel in SF from cohorts of healthy donors, patients with early- or late-stage OA, and patients with active RA.

## Methods

### Synovial fluid donors

SF samples were collected from knee joints of 16 postmortem donors, 20 patients with rheumatoid arthritis (RA), and 48 patients with OA. This study was approved by the ethical review committee of the Faculty of Medicine (Justus-Liebig-University of Giessen, Germany), and all patients provided written informed consent for their donor samples to be used for research. The ethical review committee waived the need for consent to be obtained from relatives of deceased donors, based on a judicial order that allowed an autopsy to be performed and to avoid additional emotional strain on relatives.

Detailed information about the SF donors is provided in Table 1. Based on the macroscopic appearance of the 6 cartilage surfaces—patella, trochlea, and femur and tibia from the medial and lateral sides—we used the Outerbridge classification (OU) [36] to subcategorize OA patients. To estimate OU scores for the entire knee joint, the 6 individual OU scores were added up and divided by 6 to generate an average score. OA joints were categorized as early (eOA; average OU ≤ 2; n = 26)- or late-stage OA (lOA; average OU > 2; n = 22). RA was diagnosed per the American College of Rheumatology [37]. Active arthritis in SF of RA patients was defined by the cell count of granulocytes (>1500/μl SF) in absence of bacteria and crystals (polarization microscopy) to exclude other forms of arthritis.

**Table 1:** Demographic and disease characteristics of donors.

	Postmortem donors n = 16	Patients with eOA n = 26	Patients with lOA n = 22	Patients with RA n = 20
**Age**	22 (20–26)	38 (26–56)	69 (53–74)	56 (49–72)
**female/male**	2/14	9/17	8/14	15/5
**BMI**	23.2 (21.2–25.0)	24.9 (23.7–28.1)	27.6 (26.1–30.4)	22.9 (24.2–32.6)
**CRP**	nd	0.5 (0.5–1.0)	1.5 (0.65–2.1)	9.7 (3.5–33.4)
**No. of cells**	nd	nd	nd	5800 (3763–12,788) cells/μl SF
**DAS28**	nd	nd	nd	2.63 (3.12–4.79)
**Outerbridge score**	nd	1.16 (0.33–1.58)	3.5 (2.5–3.6)	nd
**K/L-score**	nd	0 (0–1)	3 (2.75–3)	nd

Inclusion criteria: both genders, age 18–85 years, BMI <40, CRP ≤ 3 mg/L, and all CRP levels for RA. Exclusion criteria: joint infection; severe liver or kidney disease; any surgery within the last 3 months; knee joint surgery within the last 6 months; diabetes mellitus (OA); drug abuse; intra-articular treatment with hyaluronate or corticosteroid treatment within the last 3 months; HIV infection; and tumor/cancer. BMI- body mass index, CRP- C-reactive protein, DAS28- disease activity score 28, K/L-score, Kellgren-Lawrence score, nd, not determined.

### Synovial fluid samples

SF was collected from knee joints by aspiration during arthroscopy (patients with early OA cartilage damages), knee replacement surgery of late OA patients or arthrocentesis during medical exam for RA [5, 38] All SF samples were first incubated for 15 min at 37°C, passed through a 1.2-μm filter to eliminated cells and cellular debris, and centrifuged at 16,100 x *g* for 45 min at RT [5, 38]. To quench protease and phospholipase activity, 10% (v/v) inhibitor cocktail was added (PPI) [5]. The supernatants were then collected and stored at -86°C until analysis.

### Level and molecular weight distribution of HA in synovial fluid

To examine HA levels in SF, samples were initially diluted 1:40,000 in 5% Tween20 in PBS (pH 7.2–7.4) and serially diluted 1:4 three times. HA concentration in human SF was measured in triplicate per dilution by sandwich ELISA (DuoSet ELISA Development kit) according to the manufacturer’s instructions (R&D Systems, Minneapolis, USA). This kit contained recombinant human aggrecan to capture HA and biotinylated recombinant human aggrecan to detect bound HA using streptavidin-conjugated horseradish peroxidase. According to the information provided by the manufacturer, the HA ELISA detects low molecular weight (15–40 kDa), medium molecular weight (75–350 kDa), and high molecular weight (>950 kDa) forms of HA.

The molecular weight distribution of HA forms in human SF was determined in duplicate by horizontal 1% agarose gel electrophoresis, as described previously [7, 14]. In brief, SF samples without PPI were first treated with proteinase K (Roche Applied Science, Mannheim, Germany) overnight. The Lo-Ladder (30–500 kDa), Hi-Ladder (0.5–1.5 MDa), and Mega-Ladder (1.5–6.1 MDa; Hyalose, Oklahoma City, USA) markers were loaded in separate lanes as references.

Electrophoresis was performed at 50 V for 3 h. The gels were stained overnight using 0.005% Stains-All (Sigma-Aldrich, Taufkirchen, Germany) in 50% ethanol and destained with 10% ethanol for a minimum of 24 h. To visualize the bands better, the staining and destaining were occasionally repeated. The molecular weight distribution of HA forms according to MW markers being commercially available (<0.5, 0.5–1.1, 1.1–3.1, 3.1–6.1, >6.1 MDa) was determined densitometrically, with automatic subtraction of background noise, using ImageJ (National Institutes of Health).

### Analysis of lubricin in human synovial fluid

Lubricin (PRG4) concentration was measured in triplicate in SF that was stored with PPI using a custom sandwich ELISA method [7]. Briefly, a capture antibody against AA1356-1373 at the C-terminus of full-length PRG4 [39] was used to coat high-binding ELISA plates, followed by incubation with SF or PRG4 standards and detection with horseradish peroxidase-conjugated peanut agglutinin, which recognizes glycosylation in the mucin domain [40]. Purified PRG4 for the standard curve was prepared from culture medium that was conditioned with bovine cartilage explants, as described [1]; purified by Superose 6 size exclusion chromatography; verified with regard to purity by western blot; and quantified by BCA protein assay.

SF was digested sequentially with S. hyaluronidase (1 U/mL, 3 h at 37°C) and sialidase A-66 (overnight at 37°C) prior to quantification. Purified PRG4 standards were also treated with sialidase. SF samples, diluted 1:4, and PRG4 standard (320 μg/mL) were loaded and serially diluted (2X). If SF was diluted during aspiration from the joint and could not be loaded at 1:4 dilution, a higher dilution—up to approximately 15X—was used.

### Analysis of lipids in human synovial fluid

Lipids were extracted from SF samples in the presence of internal standards (Avanti Polar Lipids, Alabaster, AL, USA) [5]. Phospholipid and sphingolipid species were quantified by electrospray ionization mass spectrometry (ESI-MS/MS) as described [5]. Lipid species were annotated according to a recently proposed shorthand nomenclature [41].

### Gas chromatography

Total fatty acids (FAs) were analyzed by gas chromatography, coupled with mass spectrometry (GC-MS), as described [42]. In brief, 50 μl of SF from lOA patients was derivatized to FA methyl esters (FAMEs) in the presence of internal standards. FAMEs were separated in a highly polar BPX70 column using a GC-2010 that was coupled to a GCMS-QP2010 detector (Shimadzu, Duisburg, Germany). Quantification was performed in the selected ion monitoring mode using calibration with authentic standards.

### Statistical analysis

Analysis of variance (ANOVA) was used to assess statistically significant differences between cohorts. Subsequently, confidence intervals for pairwise differences between cohorts (control, eOA, lOA, RA) were adjusted by Tukey’s honestly significant difference (HSD) procedure. The statistical analysis was performed with R, version 3.1.0 using linear models of the logarithm or the logits of the response. The data were expressed as medians with interquartile ranges for the box plots. The values in the text are the medians with interquartile ranges in brackets.

## Results

### Level and molecular weight distribution of HA in human SF

**Fig 1:**
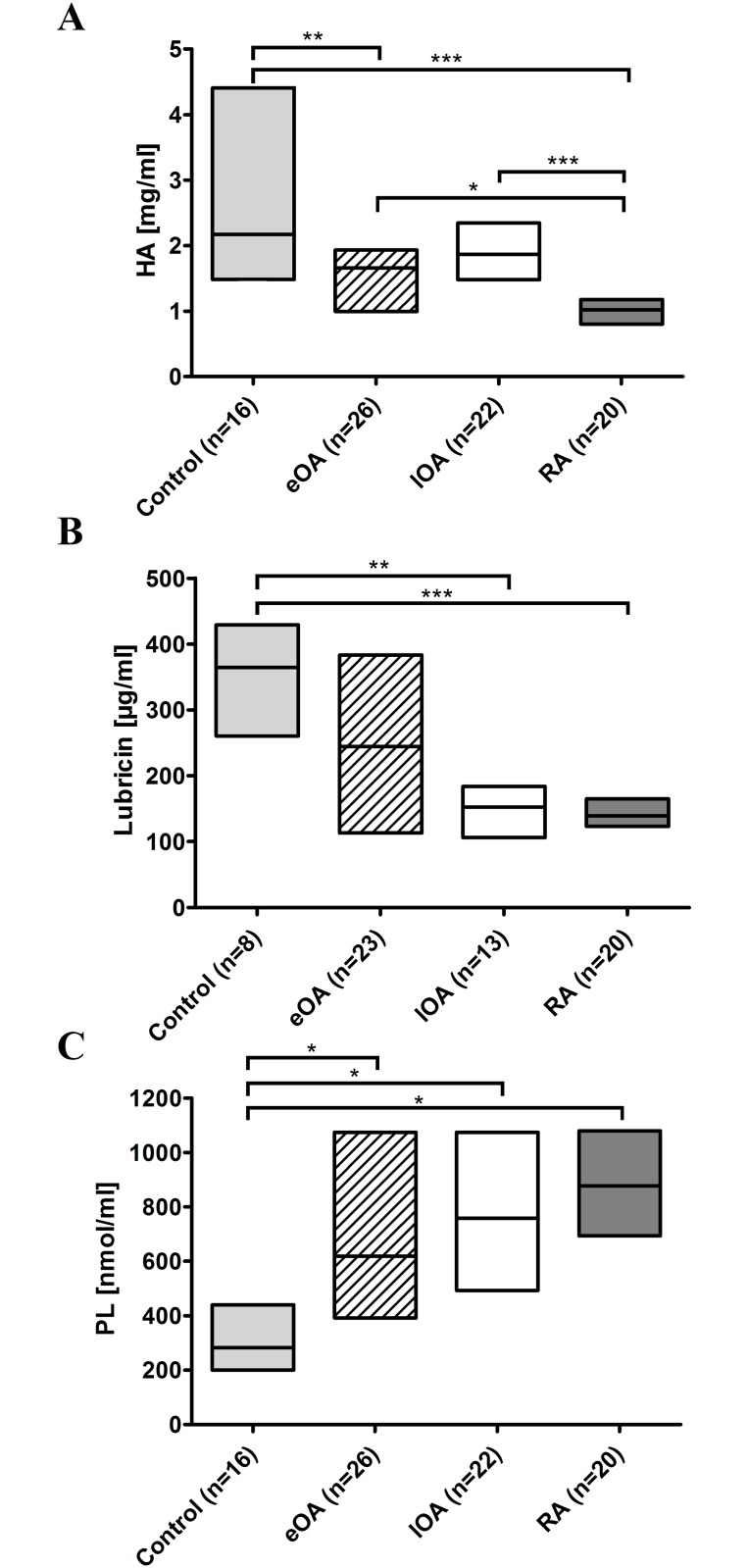
The concentrations of boundary lubricants in human SF. The content of HA (**A**) and lubricin (**B**) in SF were determined by ELISA, and ESI-MS/MS was used to quantify phospholipids (**C**) in 16 control SF (shaded bars), 27 eOA SF (hatched bars), 22 lOA SF (open bars), and 20 RA SF (grey bars) as described in Material and Methods. Data are presented as median with interquartile ranges. *P*-values less than 0.05 were considered statistically significant: *0.01< p≤0.05, **0.001<p≤0.01, ***p≤0.001.

By sandwich ELISA, as shown in Fig 1A, the concentrations of HA were highest in control SF [2.2 mg/ml (1.6–3.7 mg/ml)], whereas the levels in eOA SF [1.7 mg/ml (1.1–1.9 mg/ml), p = 0.004] and lOA SF [1.9 mg/ml (1.5–2.3 mg/ml), n.s.] were lower by 23.7% and 14.0%, respectively. The lowest concentrations were observed in RA SF [1.0 mg/ml (0.8–1.2 mg/ml), p<0.001], representing 47.1% of HA in control SF (100%).

**Fig 2:**
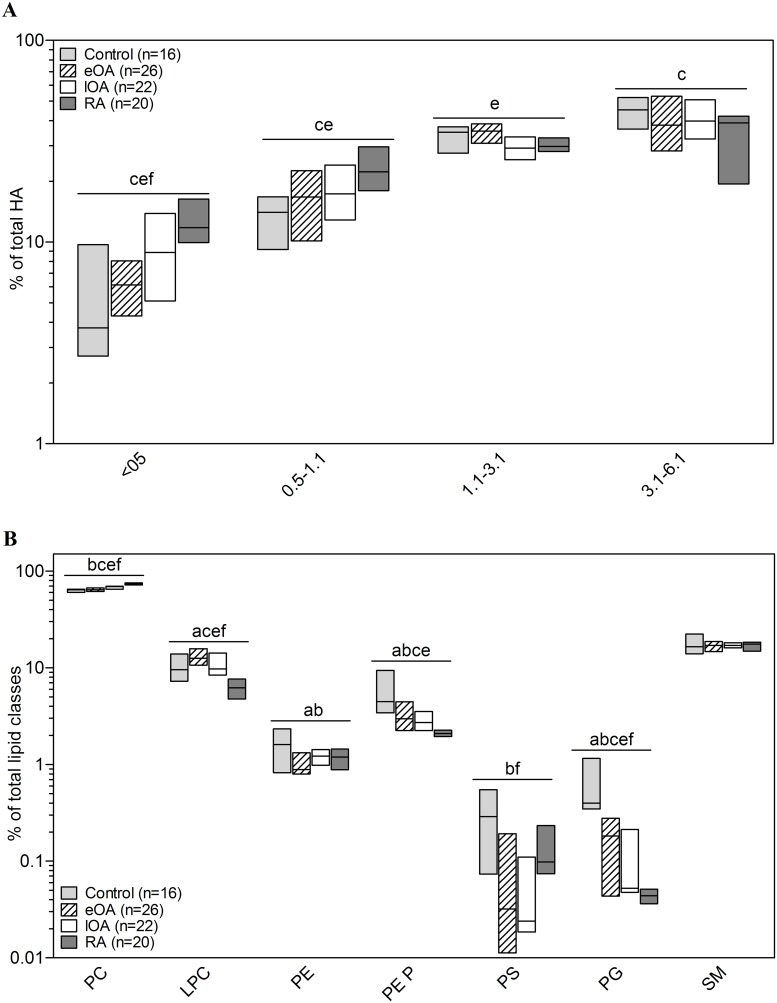
Characterization of hyaluronic acid (HA) and lipids in human SF. The molecular weight distribution of HA (**A**) was determined by horizontal agarose gel electrophoresis, and ESI-MS/MS was used to quantify phospholipid classes (**B**) in 16 control SF (shaded bars), 26 eOA SF (hatched bars), 22 lOA SF (open bars), and 20 RA SF (grey bars) as described in Methods. The molecular weight distribution of HA was calculated as the percentage of total HA (= 100%), whereas the relative distribution of phospholipid classes is shown as the percentage of total lipid content (= 100%). Data are presented as the median and interquartile ranges. Significance was defined as follows: a: p≤0.05: control vs. RA; b: p≤0.05: control vs. eOA; c: p≤0.05: eOA vs. late OA; and d: p≤0.05: eOA vs. RA, e: p≤0.05: eOA vs. RA; and f: p≤0.05: lOA vs. RA. The lipids that we measured were phosphatidylcholine (PC), lysophosphatidylcholine (LPC), phosphatidylethanolamine (PE), PE-based plasmalogens (PE P), phosphatidylserine (PS), phosphatidylglycerol (PG), and sphingomyelin (SM).

Relative HA concentration was calculated as the percentage of total HA, defined as 100%. By horizontal agarose gel electrophoresis [7, 14], compared with control SF, the MW of HA shifted toward the lower range in OA and RA SF (Fig 2A). The percentage of HA in the upper range of 3.1–6.1 MDa was highest in control SF [46.0% (39.6% to 51.2%)], followed by eOA [40.0% (28.5% to 52.7%)] and lOA SF [41.2% (32.9% to 50.7%)], and bottoming in RA SF [38.1% (21.8% to 41.8%), p = 0.03]. In the lower ranges of 0.5–1.1 MDa and <0.5 MDa, the relative HA concentrations were significantly higher in eOA, lOA, and RA SF versus control SF (p<0.001).

### Lubricin content in human SF

By ELISA, the concentration of lubricin in control SF [364.4 μg/ml (305.0–404.8 μg/ml), n = 8)] was 1.5-fold higher than in eOA [244.5 μg/ml (119.6–381.7 μg/ml), n = 23] (Fig 1B). Notably, compared with control SF, the concentration of lubricin declined by 58.2% in lOA SF [152.3 μg/ml (108.2–183.9 μg/ml), p = 0.005] and by 61.7% in RA SF (139.4 μg/ml (124.6–162.4 μg/ml), p<0.001].

### Levels of phospholipids in human SF

Lipids were extracted from cell- and cellular debris-free SF samples of 16 controls and 26 eOA, 22 lOA, and 20 active RA subjects and quantified by ESI-MS/MS. The content of total phospholipids in SF was calculated as the sum of the concentrations of all lipid species that contained a phosphate group. Compared with control SF [314.2 nmol/ml (247.3–487.1 nmol/ml)], the concentrations of phospholipids rose by 2.1-fold in eOA [643.8 nmol/ml (394.9–1106.5 nmol/ml), p = 0.03], 2.4-fold in lOA SF [758.8 nmol/ml (503.3–1009.7 nmol/ml), p = 0.01], and 2.8-fold in RA SF [877.7 nmol/ml (713.3–1065.0 nmol/ml), p = 0.015] (Fig 1C).

### Correlation between lubricants

**Fig 3:**
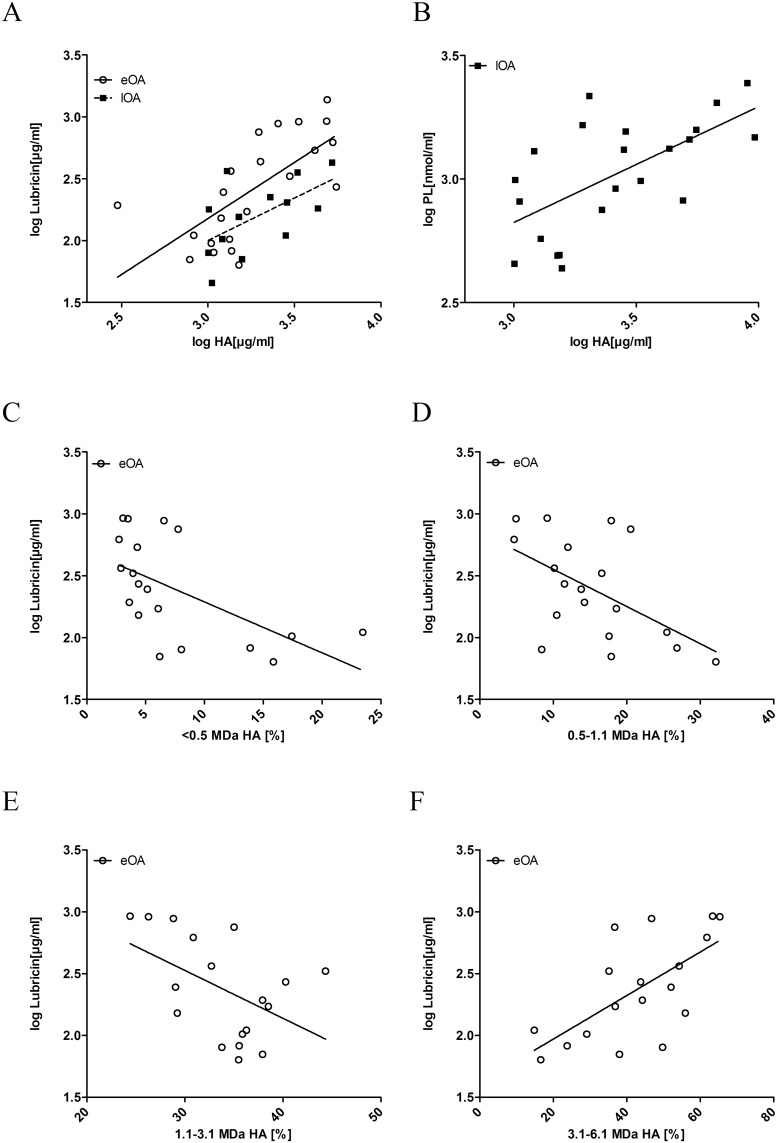
Scatterplot of concentrations of hyaluronic acid (HA) by levels of lubricin (A) and phospholipids (PL) (B) and concentration of lubricin by MW distribution of HA (C-F). HA and lubricin content in SF was determined by ELISA, and ESI-MS/MS was used to quantify phospholipids in 16 control SF, 27 eOA SF, and 22 lOA SF samples as described in Methods. Molecular weight distribution of HA was calculated as the percentage of total HA (= 100%). Linear regression was performed, and Pearson correlation coefficients were calculated.

The concentrations of all lubricants were plotted against each other to examine their correlations (Fig 3); the data were analyzed by linear regression, and the slopes, 95% confidence intervals, and Pearson correlation coefficients (r) were calculated. In eOA SF, the concentrations of HA and lubricin correlated significantly (r = 0.69, Fig 3A, p<0.001). Further, in lOA SF, correlations between the levels of HA and lubricin (r = 0.62, Fig 3A, p = 0.002) and between HA and phospholipids (r = 0.63, Fig 3B, p = 0.002) were observed.

Notably, lubricin concentration and the MW distribution of HA were associated only in eOA SF [Fig 3C (R = 0.590, p = 0.008), Fig 3D (R = 0.540, p = 0.02, Fig 3E (R = 0.486, p = 0.04), and Fig 3F (R = 0.656, p = 0.003)]. Increasing percentage of degraded HA (Fig 3C–3E) corresponded to lower levels of lubricin, whereas higher percentages of high-molecular-weight HA were linked to greater concentrations of lubricin (Fig 3F).

### Phospholipid classes and species in human SF

Seven lipid classes were identified in human SF: phosphatidylcholine (PC), lysophosphatidylcholine (LPC), phosphatidylethanolamine (PE), phosphatidylethanolamine-based plasmalogen (PE P), phosphatidylserine (PS), phosphatidylglycerol (PG), and sphingomyelin (SM) (Fig 2B). PC was the most predominant lipid class, constituting 52.7% to 80.8% of all lipids. The percentage of PC was lowest in SF from the control group [63.6% (60.7% to 64.7%)], increasing to 63.4% in eOA SF (61.6% to 66.9%), 69.0% in lOA SF (64.9% to 69.6%), and 72.9% in RA SF (71.9% to75.3%).

In contrast, compared with control SF [4.8% (3.7% to 9.1%)], the percentage of PE P declined by 39.6% in eOA SF [2.9% (2.2% to 4.4%), p<0.001], 39.6% in lOA SF [2.9% (2.3% to 3.4%), p<0.001], and 56.3% in RA SF [2.1% (2.0% to 2.3%), p<0.001]. The lowest percentages of LPC were observed in control [9.0% (7.3% to 13.8%)] and RA SF [6.5 (4.8% to 7.5%)], whereas LPC percentage peaked in eOA SF [12.7% (11.1% to 15.6%) and lOA SF [10.3% (9.1% to 13.8%), p<0.001].

**Fig 4:**
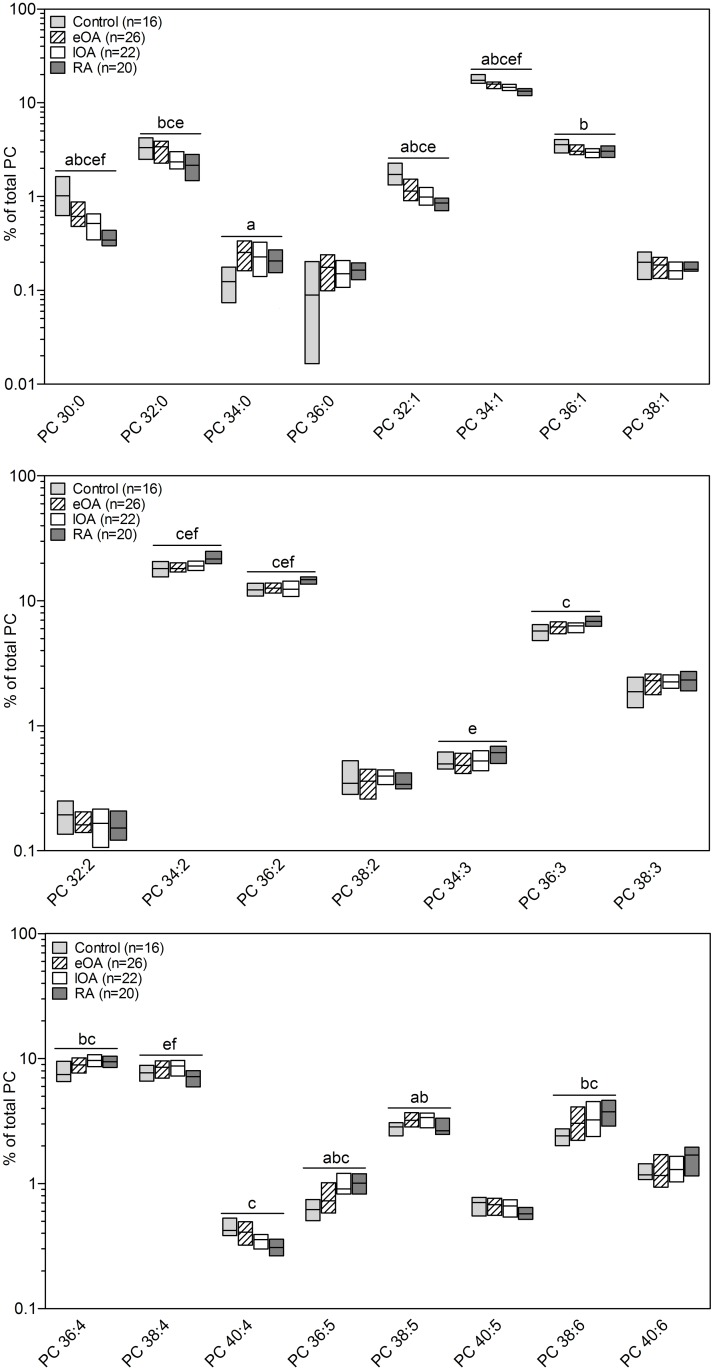
Percentage composition of phosphatidycholine (PC) species in human SF. PC species were quantified by ESI-MS/MS in 16 control SF (shaded bars), 27 eOA SF (hatched bars), 22 lOA SF (open bars), and 20 RA SF (grey bars) samples as described in Methods. Species were assigned based on the assumption that only FAs with an even number of carbon atoms are present. Subsequently, the percentage was calculated, defining total PC as 100%. Data are presented as the median and interquartile ranges. Significance was defined as follows: a: p≤0.05: control vs. RA; b: p≤0.05: control vs. eOA; c: p≤0.05: eOA vs. late OA; d: p≤0.05: eOA vs. RA, e: p≤0.05: eOA vs. RA; and f: p≤0.05: lOA vs RA.

**Fig 5:**
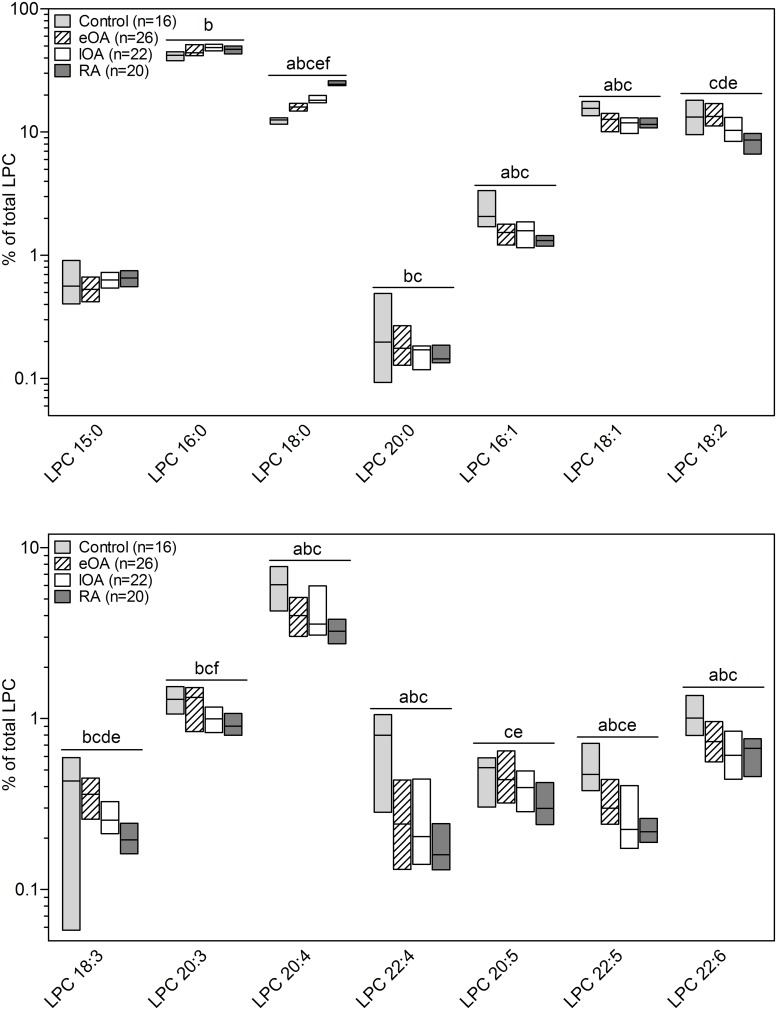
Percentage composition of lysophosphatidylcholine (LPC) species in human SF. PC species were quantified by ESI-MS/MS in 16 control SF (shaded bars), 27 eOA SF (hatched bars), 22 lOA SF (open bars), and 20 RA SF (grey bars) samples as described in Methods. Species were assigned based on the assumption that only FAs with an even number of carbon atoms are present. Subsequently, the percentage was calculated, defining total LPC as 100%. Data are presented as the median and interquartile ranges. Significance was defined as follows: a: p≤0.05: control vs. RA; b: p≤0.05: control vs. eOA; c: p≤0.05: eOA vs. late OA; d: p≤0.05: eOA vs. RA e: p≤0.05: eOA vs. RA; and f: p≤0.05: lOA vs RA.

The 7 lipid classes in human SF comprise 124 lipid species. Figs 4 and 5 shows all 23 PC and 15 LPC species. Quantitatively, PC 34:2 was the main species in the PC class, constituting 18% to 22% of total PC. The species pattern of PC was similar between all cohorts, except for PC 30:0, PC 32:1, PC 34:1, PC 34:2 and PC 36:5. For instance, when compared with control SF [17.6% (16.3% to 19.9%)] the percentage of PC 34:1 decreased in eOA SF [15.8% (14.4% to 16.7%), p<0.001], lOA SF [14.7% (13.8% to 15.6%), p<0.001], and RA SF [13.3% (12.0% to 14.0%), p<0.001]. However, the percentage of PC 36:5 rose in eOA SF [0.72% (0.58% to 0.95%), p = 0.02], lOA [0.91% (0.83% to 1.15%), p = 0.001], and RA SF [1.01% (0.85% to 1.13%), p<0.001] versus control SF [0.62% (0.51–0.71%)] (Fig 4).

The predominant LPC species in SF was LPC 16:0, accounting for 42% to 49% of all LPCs (Fig 5). The percentages of most LPC species peaked in control SF compared with other cohorts, except for LPC 18:0, which was 1.5-fold higher in lOA SF [18.1% (17.3% to 19.7%], p<0.001] and 2-fold higher in RA SF [24.4% (23.9% to 26.1%), p<0.001] than in control SF [12.5% (11.6% to 13.0%)] (Fig 5).

**Fig 6:**
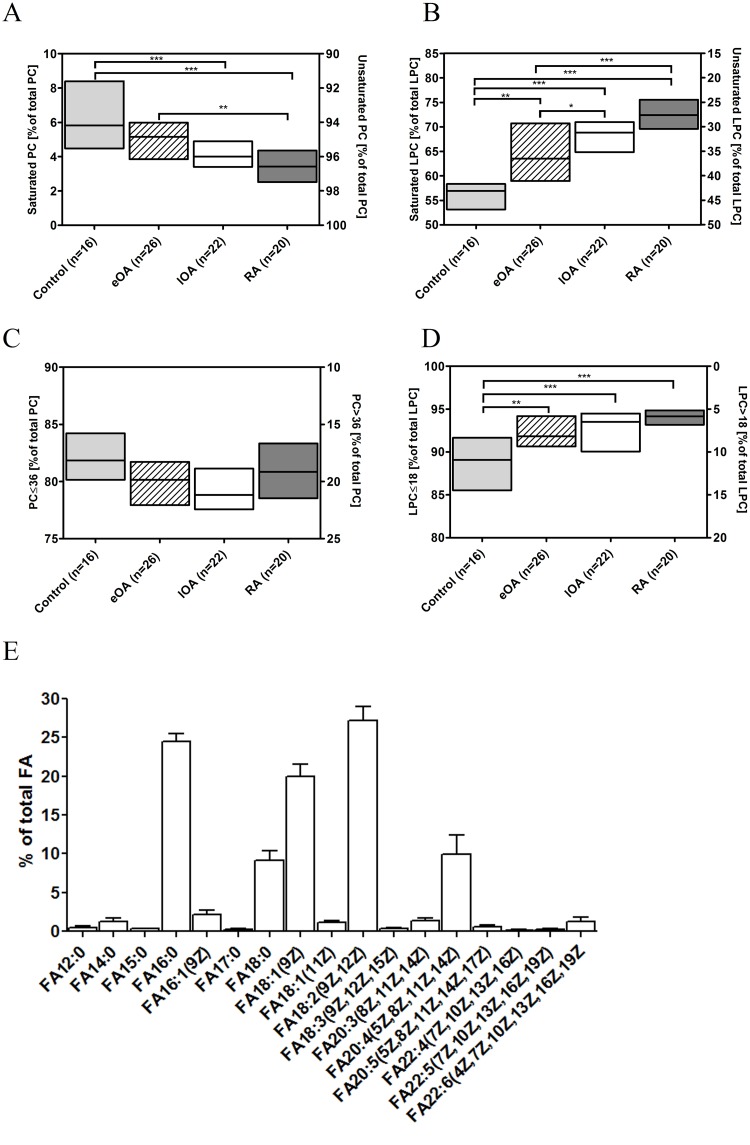
The degree of saturation and length of fatty acids from phosphatidylcholine (PC) and lysophosphatidylcholine (LPC). Phospholipid species were quantified by ESI-MS/MS in 16 control SF (shaded bars), 27 eOA SF (hatched bars), 22 lOA SF (open bars), and 20 RA SF (grey bars) samples as described in Methods. Subsequently, PC and LPC species were grouped by degree of saturation and number of carbon atoms in the FA chains, respectively, and calculated as a percentage of total PC and LPC, defined 100%. Composition of total FA was determined by gas chromatography for 6 lOA SF samples as described in Methods. Data are presented as median and interquartile ranges **(A-D)** or mean ± SD **(E)**. *P*-values less than 0.05 were considered statistically significant: *0.01< p≤0.05, **0.001<p≤0.01, ***p≤0.001. **A,** Relative distribution of PC species according to degree of FA saturation. **B,** Relative distribution of LPC species by degree of FA saturation. **C,** Relative distribution of PC species according to chain length of FAs. **D,** Relative distribution of LPC species by chain length of FAs. **E,** Degree of saturation and length of fatty acid of total FAs representing 100%.

The biophysical properties of lipid species depend on the fatty acid (FA) chain length and the degree of FA saturation. Thus, we calculated the relative contribution of PC and LPC species with various FA chain lengths and degrees of saturation to the total amount of PCs and LPCs (Fig 6). PC contains 2 FAs, but our mass spectrometry method only analyzed the sum of carbon atoms and double bonds in FAs.

In all cohorts, most PCs were unsaturated (Fig 6A)—7.2% of PC species in control SF, 5.1% in eOA SF, 4.1% in lOA SF, and 3.6% in RA SF were present in saturated form (p<0.001). In contrast, FAs were more saturated among LPCs (Fig 6B). Compared with control SF, [56.9% (53.4% to 58.3%)], more LPC species with saturated FAs were recorded in eOA [63.5% (59.0% to 70.7%), p<0.001], lOA [68.8% (65.3% to 70.6%), p<0.001], and RA SF [72.4% (70.2% to 75.4%), p<0.001].

Analysis of FA chain lengths revealed that more than 80% of PC species contained 36 C-atoms or less and that nearly 20% of PC species more than 36 C-atoms (Fig 6C), but these differences were not significant between cohorts. In all cohorts, approximately 90% of all LPC species had FA chain lengths with 18-C atoms or less. Moreover, versus control SF, [89.1% (86.2% to 91.5%)], the lengths of FA chains of LPC species were shorter in eOA [91.8% (90.7% to 94.0%), p<0.001], lOA [93.5% (90.3% to 94.4%), p<0.001], and RA SF [94.2% (93.2% to 94.8%), p<0.001] (Fig 6D).

In order to get an overview of the FAs in SF, total composition was determined by gas chromatography coupled to mass spectrometry (GS-MS) for 6 lOA SF samples (Fig 6E). The principal FAs were FA 16:0, FA 18:1, and FA 18:2, each of which accounted for 20–25% of total FAs. The polyunsaturated FAs constituted 13.5%. Medium long (<14 C-atoms) and very long (22–24 C atoms) FA chains were minor FAs [1.72% (1.26% to 1.92%) and 1.69 (0.91% to 2.12%), respectively].

### Lubricants and age dependency

Our cohorts differ with respect of their age (Table 1). Thus, the levels of lubricants were plotted against the age of each donor within each cohort (S1 Fig). Subsequently, linear regression was performed, and Pearson correlation coefficients were calculated. We did not find any age dependency on the levels of all three lubricants (S1 Fig, [43]). In addition, the HA MW distributions were plotted against the age. Again, our analysis revealed no correlation between the HA MW distribution in SF of our four cohorts and the age of donors (S2 Fig). We already reported that no correlation was found between the levels of PLs and the age of patients [5].

## Discussion

HA, lubricin, and phospholipids contribute to cartilage boundary lubrication that is provided by SF [1, 3, 4]. However, the most important SF component in maintaining efficient joint lubrication has long been debated. This study is the first to measure the levels of HA, lubricin, and phospholipids in SF from the same cohorts of healthy donors, patients with early- and late-stage OA, and patients with active RA. Further, we have detailed the MW forms of HA, the relative distribution of phospholipid classes, the phospholipid species composition, and the degree of saturation and FA chain lengths.

OA and RA SF contained less HA and lubricin and are enriched with phospholipids. Also, the MW distribution of HA shifted toward the lower range in OA and RA SF. These results indicate that the catabolic activities in OA and RA SF are enhanced, leading to decreased levels of lubricin and high-MW HA.

The levels of lubricin vary in OA SF compared with control SF. Some studies have demonstrated lower levels of lubricin in OA and RA SF [1, 44–46], whereas others have reported opposite results [8, 20, 44]. In our study, the levels of lubricin are consistent with those of Ludwig et al., who observed that some OA SF is lubricin-deficient and has poor boundary-lubricating ability compared with healthy control SF [7]. Further, we confirmed our previous findings that phospholipid levels are elevated under pathological conditions, which might compensate for the decreased levels of HA and lubricin in SF to protect cartilage surfaces against wear [5, 43].

Several studies have attempted to characterize the components of SF, but none has determined which species is crucial for effective cartilage boundary lubrication. HA provides viscosity in SF and mediates the retention of water [9]. Lubricin has been suggested to be the most important component for cartilage boundary lubrication and has chondroprotective properties [3]. Phospholipids are believed to cover the surface of articular cartilage, where they generate a microscopically thick biofilm, contributing to cartilage boundary lubrication [4, 30]. However, the interaction between these components and their functions in cartilage boundary lubrication remain poorly understood.

Several models of cartilage boundary lubrication have been proposed. A model that was developed by Hills claims that a mono- to multilayered membrane-like structure adheres to the surface of articular cartilage, where HA and phospholipids interact and form stable complexes. HA molecules constitute a core filament that is surrounded by a bilayer of phospholipids, generating oligolamellar structures [30, 47]. Another model opines that a network of multilamellar vesicles exists—by transmission electron microscopy and biotribological techniques, there are vesicular structures that comprise 3–7 lipid bilayers that encapsulated HA with seric proteins, such as albumin and y-globulin. Here lubricin functions solely to anchor this lipid layer to the articular surfaces [2]. We noted correlations between the concentrations of HA and lubricin, between the levels of HA and phospholipids, and between lubricin levels and the HA MW distribution in SF, supporting a model of tight interactions between these molecules.

The friction-reducing properties of HA at the cartilage-cartilage interface depend on the concentration and molecular weight of HA [6, 44]. High-MW HA has distinct cartilage lubricating abilities, and *in vitro* supplementation with high-MW HA is believed to partially restore the cartilage boundary lubricating function of SF [44]. We found that OA and RA SF contain significantly higher levels of small-MW HA, mitigating the impact of HA on lubrication. However, HA in the MW range of 0.5–1.1 x 10(6) Da was reported to restore SF rheological properties in animal models of OA (13).

The phospholipid organization on the surface of articular cartilage is thought to depend strictly on the MW of HA [47]. Throughout the enzymatic degradation of HA during joint inflammation and OA, a membrane-like sheet that is constructed from phospholipids is destroyed. Thus, altered MW distribution of HA can impair cartilage boundary lubrication and might also explain the elevated levels of phospholipids in OA and RA SF.

The lubricating properties of phospholipids remain poorly understood. However, the tribological properties of phospholipids depend on the molecular structure of the lipids—especially chain length and the number of double bounds. To better understand the molecular mechanisms of the lubricating abilities of phospholipids, the pattern of phospholipid species must be analyzed in details. Dipalmitoylphosphatidylcholine has been considered to be the chief phospholipid in SF, as it is in the lung [30]. However, we and other groups have shown that unsaturated forms of PC, such as dioleoylphosphatidylcholine, palmitoyloleoylphosphatidylcholine, and palmitoyllinoleoylphosphatidylcholine, are the predominant phospholipids in SF [5, 48].

Further, LPC chain length is significantly shorter in OA and RA SF compared with control SF. One study on boundary lubrication found that shorter FA chains increase the friction coefficient, whereas more extensive unsaturation reduces the friction [49]. Further studies are needed to elaborate the overall lubricating ability of these altered PC and LPC species in SF.

## Conclusions

In conclusion, we have provided novel comprehensive measurements of HA, lubricin, and phospholipids levels in SF from the same cohorts of healthy donors, patients with early- and late-stage OA, and patients with RA. Our study provide insight into the detailed composition of SF, correlating the concentrations of lubricants and the alterations that occur during widespread joint diseases, which can impair the joint lubricating ability of SF. These results extend our current knowledge on cartilage boundary-lubricating molecules. Thus our study provides the framework to develop new optimal compounded lubricants able to reduce joint destruction by improved lubrication.

## Supporting Information

S1 Fig Scatterplot of lubricin and hyaluronic acid (HA) levels by the age of donors. HA and lubricin content in SF was determined by ELISA in 16 control SF **(A)**, 27 eOA SF **(B)**, 22 lOA SF **(C)**, and 20 RA SF samples **(D)** as described in Methods. Linear regression was performed, and Pearson correlation coefficients were calculated. The Pearson correlation coefficients (r) were as follow: r = -0.12 for HA and r = 0.33 for lubricin in control SF; r = -0.29 for HA and r = -0.19 for lubricin in eOA SF; r = -0.59 for HA and r = -0.45 for lubricin in lOA SF; r = 0.23 for HA and r = -0.01 for lubricin in RA SF.
(TIF)

S2 Fig Scatterplot of the hyaluronic acid (HA) molecular weight distribution by the age of donors. HA content in SF was determined by ELISA in 16 control SF **(A)**, 27 eOA SF **(B)**, 22 lOA SF **(C)**, and 20 RA SF samples **(D)** as described in Methods. The molecular weight distribution of HA was calculated as the percentage of total HA (= 100%). Linear regression was performed, and Pearson correlation coefficients were calculated. The Pearson correlation coefficients range as follow: from -0.30 to 0.10 for control SF; from -0.10 to 0.07 for eOA SF; from -0.11 to 0.19 for lOA SF, and from -0.10 to 0.14 for RA SF.
(TIF)
