# Supplementary figures and images for: Articular Joint Lubricants during Osteoarthritis and Rheumatoid Arthritis Display Altered Levels and Molecular Species
Source: PLoS One. 2015 May 1;10(5):e0125192. doi: 10.1371/journal.pone.0125192 (PMC4416892; doi:10.1371/journal.pone.0125192)

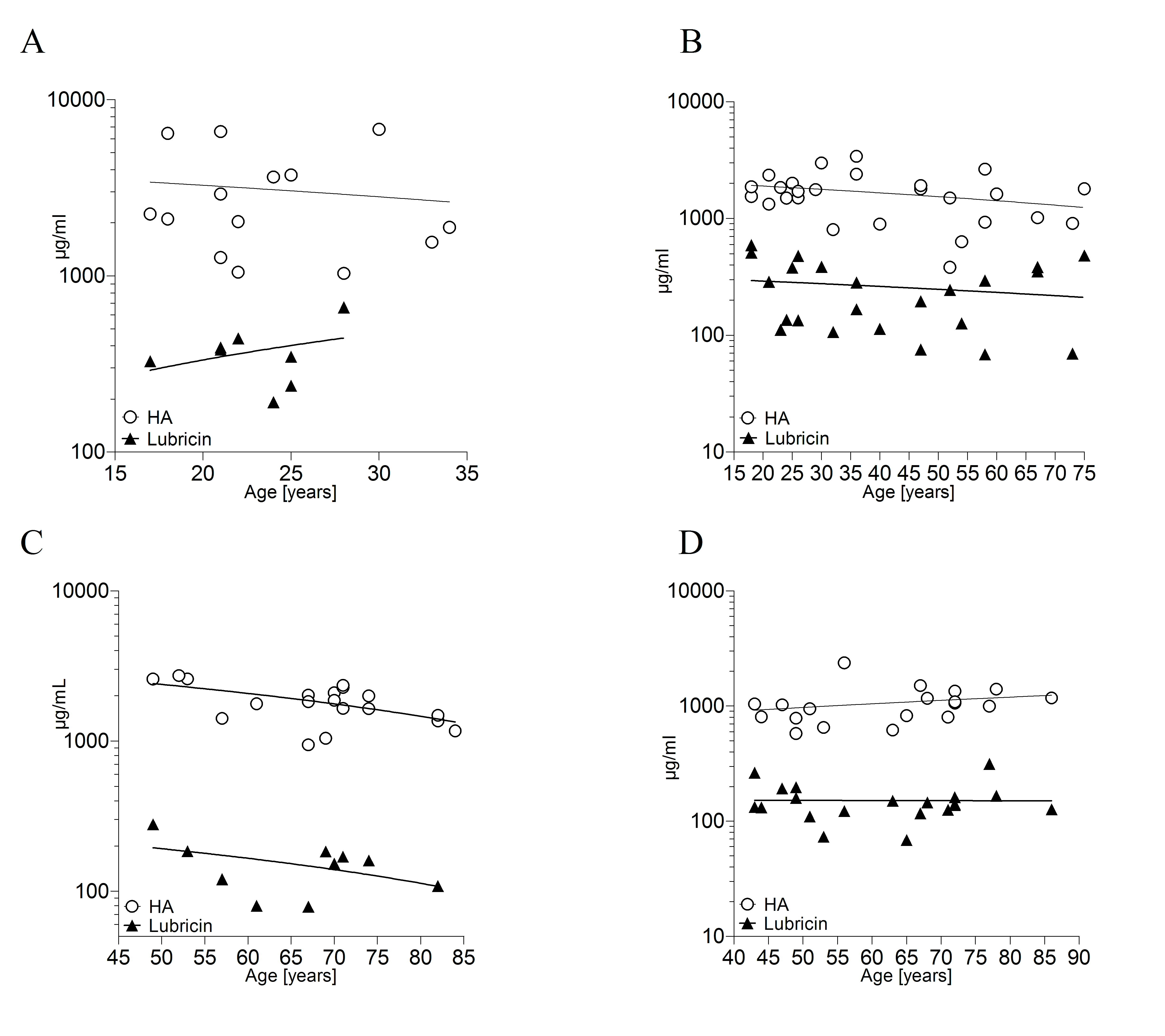

Supplement: S1 Fig — HA and lubricin content in SF was determined by ELISA in 16 control SF (A), 27 eOA SF (B), 22 lOA SF (C), and 20 RA SF samples (D) as described in Methods. Linear regression was performed, and Pearson correlation coefficients were calculated. The Pearson correlation coefficients (r) were as follow: r = -0.12 for HA and r = 0.33 for lubricin in control SF; r = -0.29 for HA and r = -0.19 for lubricin in eOA SF; r = -0.59 for HA and r = -0.45 for lubricin in lOA SF; r = 0.23 for HA and r = -0.01 for lubricin in RA SF. (TIF) [file pone.0125192.s001.tif]

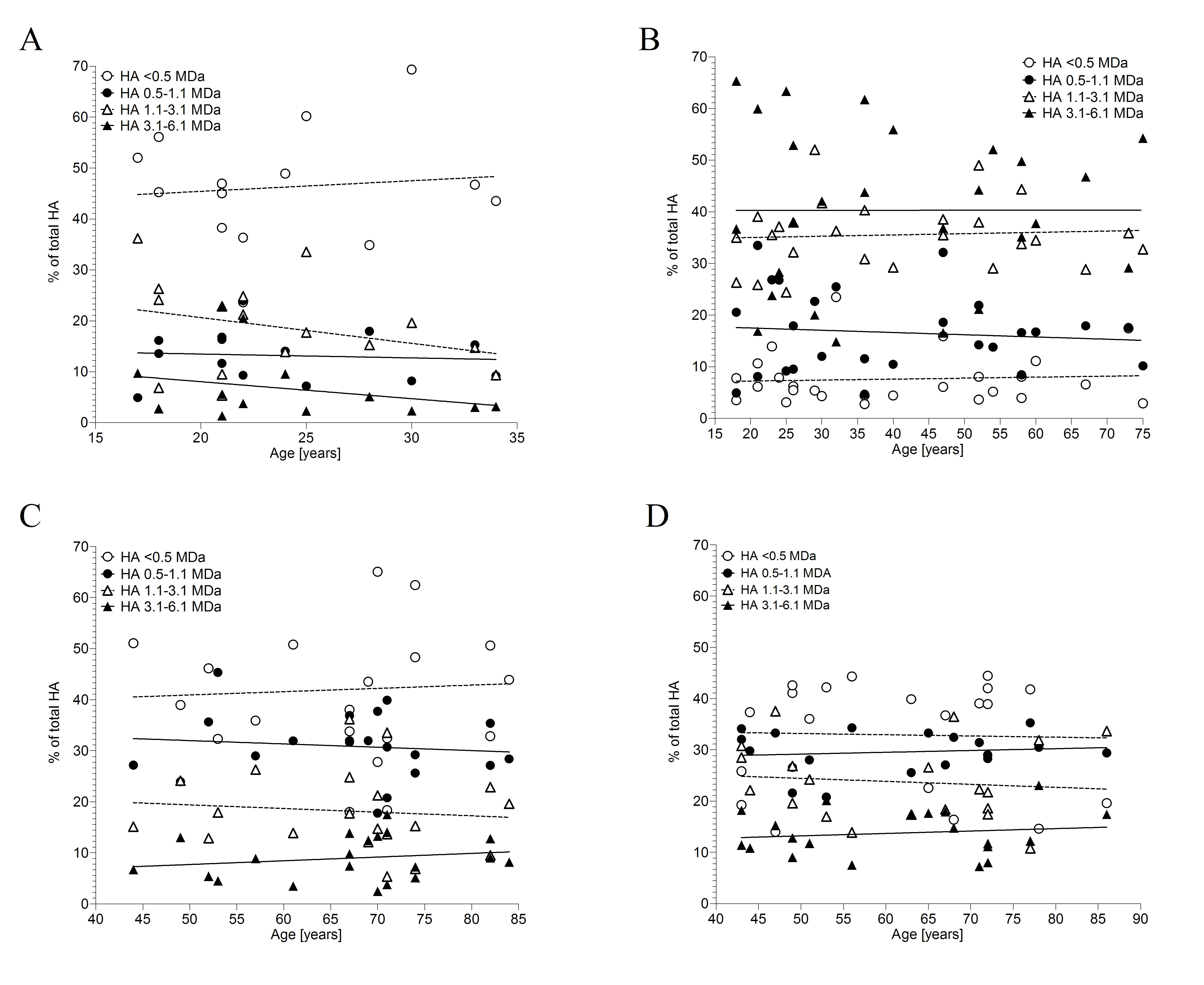

Supplement: S2 Fig — HA content in SF was determined by ELISA in 16 control SF (A), 27 eOA SF (B), 22 lOA SF (C), and 20 RA SF samples (D) as described in Methods. The molecular weight distribution of HA was calculated as the percentage of total HA (= 100%). Linear regression was performed, and Pearson correlation coefficients were calculated. The Pearson correlation coefficients range as follow: from -0.30 to 0.10 for control SF; from -0.10 to 0.07 for eOA SF; from -0.11 to 0.19 for lOA SF, and from -0.10 to 0.14 for RA SF. (TIF) [file pone.0125192.s002.tif]
